# Matrix metalloproteinase 9 expression in primary human prostatic adenocarcinoma and benign prostatic hyperplasia.

**DOI:** 10.1038/bjc.1994.30

**Published:** 1994-01

**Authors:** F. C. Hamdy, E. J. Fadlon, D. Cottam, J. Lawry, W. Thurrell, P. B. Silcocks, J. B. Anderson, J. L. Williams, R. C. Rees

**Affiliations:** Department of Urology, Royal Hallamshire Hospital, Sheffield, UK.

## Abstract

**Images:**


Br. J. Cancer (1994), 69, 177-182 © Macmillan Press Ltd., 1994

### Matrix metalloproteinase 9 expression in primary human prostatic adenocarcinoma and benign prostatic hyperplasia

F.C. Hamdy[1,3], E.J. Fadlon[3], D. Cottam[5], J. Lawry[3], W. Thurrell[2], P.B. Silcocks[4], J.B. Anderson[1], J.L. Williams[1] & R.C. Rees[3]

*Departments of [1]Urology and [2]Pathology, Royal Hallamshire Hospital, Sheffield S10 2JF; [3]Institute for Cancer Studies and [4]Department of Public Health Medicine, University of Sheffield Medical School, Sheffield S10 2RX, UK; [5]Department of Health and Human Services, Bethesda, 900 Rockville Pike, Maryland, USA.*

**Summary** Matrix metalloproteinase (MMP) expression was investigated in patients with prostatic adenocarcinoma and benign prostatic hyperplasia (BPH). Forty-one men were studied: 26 had histologically proven prostate cancer, with 14 (54%) showing metastatic disease; 15 patients had BPH. Prostatic tissue was obtained from transurethral resection and needle core biopsies; gelatinolytic activity was determined by zymography. Seven gelatinolytic bands were detected, with molecular weights ranging from > 100 kilodalton (kDa) to 29 kDa. Nine of 14 patients (64%) with skeletal metastases had 92 kDa activity, present in only two of 12 patients (17%) with a negative bone scan, and absent in BPH. The 92 kDa gelatinolytic activity was expressed in 73% of aneuploid tumours compared with 20% of diploid tumours. A 97 kDa gelatinase was expressed in 80% of BPH samples and 23% of carcinoma patients. Enzyme bands of 72, 66 and 45 kDa were equally expressed in malignant tissue, irrespective of metastatic status, but were expressed in fewer BPH patients. The 97, 92, 66 and 45 kDa enzymes were identified as being pro-MMP-9 sequences by Western blotting, using a specific antibody directed against the pro sequence of the mature protein. MMP activity appeared to be increased in malignant prostatic tissue compared with BPH. Pro-MMP-9, in its 92 kDa form, was shown to be exclusively expressed by malignant prostatic tissue, and in particular by tumours that exhibited the aggressive and metastatic phenotype.

Prostate cancer is the third most common malignancy in men in England and Wales, with over 9,000 new cases registered every year (Office of Population Censuses and Surveys, 1985). Approximately 50% of patients present with metastatic disease and have a poor prognosis, with a median survival of less than 3 years (Whitmore, 1984). By contrast, a 5-year survival rate of up to 93% can be expected in those patients with localised disease which fails to progress beyond the confines of the gland (George, 1988; Johansson *et al.*, 1989, 1992). Autopsy studies have also shown that many men over the age of 80 harbour a prostatic cancer which never becomes clinically manifest (Hirst & Bergman, 1954). The reason for such discrepancies in the presentation and subsequent behaviour of prostate cancer remains unclear, and represents a great challenge to clinicians and scientists.

Attempts have been made to predict the biological behaviour of prostate cancer. While tumour staging and volume, serum prostate-specific antigen (PSA) measurements, histopathological grading and deoxyribonucleic acid (DNA) tumour ploidy status have all be shown to correlate with prognosis and survival (Tavares *et al.*, 1973; de Vere White & Deitch, 1989; Gittes, 1991), no single method can reliably distinguish between potentially progressing tumours and those which will remain quiescent. New reliable criteria are thus needed to define the invasive and metastatic potential of individual prostate cancer cases. This has prompted the present research to be undertaken into the potential value of matrix metalloproteinase (MMP) expression in predicting metastatic disease in prostate cancer.

To date, five subgroups of MMP have been identified (MMP-1 and -8, MMP-3 and -10, MMP-7, MMP-2 and -9, MMP-11), depending on the relationship of each enzyme to bacterial zinc-containing proteinases (Murphy *et al.*, 1991; Woessner, 1991; Cottam & Rees, 1993). The MMP family can also be divided into three subclasses according to homology and substrate specificity; these are the gelatinases (type IV collagenases), the stromelysins and the interstitial collagenases (Matrisian, 1990). MMPs are involved in both physiological processes, including embryonic development, post-partum uterine involution, ovulation and wound healing, and in pathological conditions, including rheumatoid arthritis and tumour invasion. Some members of the MMP family of enzymes are capable of degrading basement membrane (BM) at the tumour–host interface, with variable enzyme production correlating with the metastatic phenotype (Liotta, 1986; Murphy *et al.*, 1989). Expression of these enzymes is now accepted as a universal important step in the metastatic cascade of events of a primary malignant tumour. Furthermore, the activity of MMP in tissues is regulated by a group of specific inhibitors: the tissue inhibitors of metalloproteinases (TIMP-1 and TIMP-2). It is the fine balance between MMP and TIMP expression which will determine, in part, the invasive ability of a given cancer cell (Liotta & Stetler-Stevenson, 1991; Liotta *et al.*, 1991).

Several reports have recently been emerging in the literature, correlating MMP expression and tumour invasiveness in prostate cancer. Using Northern blotting to study MMP gene expression in benign and malignant prostatic tissue, Pajouh *et al.* (1991) found that MMP-7 was increased in malignant compared with benign prostatic tissue, but absent in the stroma; and that type I collagenase and stromelysins were also present in prostate cancer tissue. Boag and Young (1993) found increased levels of the 72 kDa type IV collagenase in malignant prostate and metastatic tissue by immunohistochemistry. Stearns and Wang (1993) analysed BPH and primary prostate cancer tissue extracts for type IV collagenase ($M_r$ 72,000) expression, using collagenase antibodies and Northern blot studies. Their results suggested that the enzyme is selectively overexpressed by malignant and preinvasive epithelial cells, with low levels in benign tissue and the stroma surrounding tumour foci. Powell *et al.* (1993) have demonstrated increased invasiveness of the prostate cancer cell line DU-145 following transfection with Matrilysin metalloproteinase (MMP-7). A more recent study by Wilson *et al.* (1993) investigated gelatinolytic and caseinolytic proteinase activities in human prostatic secretions, showing a variety of proteinase activity with differential levels of expression in neoplastic compared with benign disease.

In the present study, zymography and Western blotting were used to investigate MMP gelatinolytic activity, and to compare MMP-9 expression patterns in tissue extracts from prostate cancer and BPH; the findings were correlated with

Correspondence: F.C. Hamdy, Department of Urology, Freeman Hospital, High Heaton, Newcastle upon Tyne, NE7 7DN, UK.

clinical data collected from each patient. The results and the possible role of MMP-9 expression in identifying potentially progressing tumours are discussed.

### Patients and methods

### *Patients*

Forty-one patients were studied. Their age ranged from 46 to 87 years (median 68 years). Fifteen patients had BPH and 26 had histologically proven prostatic adenocarcinoma. All patients had three serial serum prostate-specific antigen (PSA) measurements (immunometric radioimmunoassay, CIS, UK) prior to prostatic manipulation, and patients with prostate cancer had a technetium-99m bone scan prior to commencement of the study. Patients with metastatic disease were treated by hormonal manipulation, in the form of bilateral orchidectomy or administration of a luteinising hormone-releasing hormone (LHRH) analogue. Patients with disease apparently localised to the prostate were treated with external beam irradiation. The follow-up period ranged from 4 to 18 months (median 10 months).

### *Sample preparation*

Prostatic tissue was obtained from transurethral resection specimens. Presence of carcinoma was confirmed by using standard histological criteria and the combined Gleason system of scoring 1 to 10 (Gleason, 1966; Gleason & Mellinger, 1974). Each tissue sample was collected in a sterile plastic container, placed on ice and transported to the laboratory within 30 min. Specimens were minced into 1 mm cubes with crossed scalpels. Representative diced samples were taken for histological examination by one observer (W.T.), and for DNA analysis. DNA ploidy status was measured by flow cytometry (Ortho Diagnostic Orthocyte), using propidium iodide (DNA CycleTest, Becton-Dickinson, UK) on at least 10,000 cells per sample of one million cells, after filtration and mechanical dissociation to obtain single-cell suspensions. DNA ploidy and cell cycle measurements were made using the Multicycle DNA Analysis Computer Package (Phoenix Flow Systems, USA) with samples excluded if coefficients of variations (CV) were greater than 10. Remaining specimens were placed in glass homogenisers in a threefold weight/volume of lysis buffer consisting of 50 mM Tris–HCl pH 7.4 with 200 mM sodium chloride and 0.1% Triton X-100. Homogenised samples were then centrifuged, supernatant pipetted off and used immediately for zymography. Protein content of each sample was determined by the modified Lowry method (Wang & Smith, 1975), and ranged from 2.3 to 3 $\mu$g per sample (average 2.5 $\mu$g).

### *Zymography*

Gelatinolytic enzyme species were detected using zymography as previously described (Heussen & Dowdle, 1980). Supernatant samples were electrophoresed (200 volts for 36 min at room temperature) in 7.5% polyacrylamide resolving gels containing 1 mg ml$^{-1}$ gelatin, using Bio-Rad Mini Protean II equipment (Bio-Rad, Richmond, CA, USA). The class of gelatinolytic proteinases was determined by addition of specific proteinase inhibitors into incubation buffer: 10 mM ethylenediaminetetraacetic acid (EDTA) for metal ion-dependent gelatinases, e.g. metalloproteinases, and 2 mM phenylmethylsulphonylfluoride (PMSF) for serine proteinases. Apparent molecular weight values were determined by comparison with reduced molecular weight markers prepared by addition of 2.5% 2-mercaptoethanol to the sample buffer, followed by boiling for 3 min. Molecular weight markers appeared as dark bands against a blue background, and consisted of carbonic anhydrase (29 kDa), egg albumin (45 kDa), bovine serum albumin (66 kDa) and phosphorylase b (97 kDa) at a concentration of 1 mg ml$^{-1}$ (Sigma, Poole, UK).

Twenty microlitres of sample supernatant, in a buffer solution containing 0.25 M Tris–HCl pH 6.8, 0.4% sodium dodecylsulphate and 34% glycerol, was loaded onto gels. After electrophoresis, gels were washed with 2% Triton X-100 for 1 h, followed by two 5 min washes in 50 mM Tris–HCl pH 7.4 containing 200 mM sodium chloride and 5 mM calcium chloride, then incubated for 48 h at 37°C. Enzymatic degradation was detected by staining gels with 0.1% (w/v) solution of Coomassie blue R250 in methanol – acetic acid – water (3:1:6). Enzyme activity was visualised as transparent bands against a blue background. After initial detection of different molecular weight bands of activity, gelatinolytic enzymes were activated in repeat experiments, incubating the samples with 1 mM para-aminophenylmercuric acetate (*p*-APMA) for 4 h at 37°C prior to zymography. *p*-APMA, an organomercurial compound, activates BM-degrading MMPs to the active lower molecular weight form by cleavage of the propeptide fragment from the amino terminus (Stetler-Stevenson *et al.*, 1989).

### *Preparation of MMP-9-specific antibody*

Antibodies specific for unique amino acid sequences present in the propeptide domains of the MMP-9 molecule were generated in-house and prepared as follows. A 20-mer peptide selected from human 92 kDa gelatinase (aa) was synthesised and purified by reversed-phase high-performance liquid chromatography on a $C_{18}$ column. Where indicated a cysteine was added to the carboxy terminus of the peptide for ease of coupling to the carrier protein. The peptide was conjugated to keyhole limpet haemocyanin (KLH) (Sigma). Free KLH was separated from KLH–peptide following chromatography on a G-25 column. The polyclonal antipeptide antibody was produced in New Zealand White rabbits using Freund's complete adjuvant. Antibody production protocols were performed according to UK Home Office regulations. The immunoglobulin G (IgG)-containing fraction was purified using Affi-gel Blue DEAE-cellulose (Bio-Rad, UK), followed by chromatography on a protein A-Sepharose column. The amino acid sequence prepared for MMP-9 was:

RQRQSTLVLFPGDLRTN

The antibody was shown to be specific for the prosequence of MMP-9. By immunoblotting, this antibody did not cross-react with the active MMP-9 enzyme, MMP-2 pro- and active enzyme, or other enzyme species.

### *Immunoblotting*

Reduced and non-reduced protein samples were separated on 7.5% acrylamide gels followed by transfer onto PVDF (polyvinylidenedifluoride) membranes (Bio-Rad, UK), following previously described protocols (Laemmli, 1970; Towbin *et al.*, 1979). Tris-buffered saline (TBS) (BioRad, UK) equilibrated membranes were blocked for 2 h in 5% non-fat milk–TBS (0.05% v/v) – Tween 20 (BioRad, UK), followed by overnight incubation with 5% non-fat milk–TBS (0.05% v/v) – Tween 20 and rabbit anti-human primary antibody, which recognised the prosequence of mature MMP-9, as described above. Subsequent steps were carried out with a BioRad Western Blot detection kit using the enclosed protocol.

### *Statistical analysis*

Statistical values were obtained using the chi-squared ($\chi^2$) test (Stat-X-ACT, Cytel Software, Cambridge, MA, USA). An adjustment was made for multiple comparison by comparing paring all *P*-values with a critical value of 0.0042 rather than the usual 0.05. This value was obtained by a modified Bonferroni method in which, instead of dividing the *P*-value by the number of significance tests performed, it is divided by an estimate of the number of true null hypotheses tested. The latter was found to be approximately 12, using the graphical method of Schweder and Spjotvoll (1982).

### Results

Technetium-99 m isotope bone scans showed increased uptake in 14 of 26 patients with prostate cancer, confirming the presence of skeletal metastases in 53.8% of the cancer population studied. DNA ploidy of malignant prostatic tissue, measured by flow cytometry, showed that 11 of 26 patients (42%) had aneuploid primary tumours; the remaining tumours were diploid. Five patients with locally advanced and metastatic disease failed to respond to treatment in the form of hormonal manipulation and died within 6 months of commencement of the study. The remaining patients showed a good immediate response to treatment, with symptomatic improvement and reduction in serum PSA levels.

Using supernatants prepared from BPH or prostatic adenocarcinoma tissue lysates, a maximum of seven gelatinolytic bands were detected by zymography, ranging from 29 kDa to >100 kDa. A summary of the patterns of expression in the different groups of patients and statistical significance is shown in Tables I and II.

The high molecular weight band (>100 kDa) of enzyme activity was predominantly present in patients with a negative bone scan (75%) and in only 36% of patients with BPH. While the 97 kDa gelatinolytic band was mostly present in BPH samples (80%) and in an average 23% of malignant tissue ($P<0.001$), the 92 kDa band was absent from BPH tissue on zymography, but present in, on average, 42% of cancer patients ($P<0.004$), 64% of patients with a positive bone scan and 17% of those without metastases ($P<0.05$) (Figure 1). Enzyme activities at molecular mass ($M_r$) 72, 66 and 45 kDa were expressed in 77% of carcinomas but only 27% ($P<0.004$), 20% ($P<0.001$) and 33% ($P<0.01$) of BPH samples respectively. A 29 kDa band of activity was present in a minority of patients with carcinoma, irrespective of their metastatic status, but was absent in BPH patients. All degradation bands appearing in gelatin zymograms were inhibited by EDTA, unaffected by PMSF and showed a decrease in molecular weight following *p*-APMA activation, suggesting that enzymes detected belonged to the MMP family.

When enzyme activities obtained by gelatin zymography were correlated with primary tumour ploidy (Table III), as measured by flow cytometry, it became apparent that the 92 kDa band of enzyme activity was present in 73% of patients with aneuploid tumours compared with only 20% of patients with diploid tumours. The other bands of enzyme activity were equally expressed in both groups, apart from the high molecular weight enzyme, which was present in nearly three times as many diploid tumours as aneuploid tumours (statistically not significant). Expression of the 92 kDa gelatinase activity also correlated with other clinical parameters, such as serum PSA levels, Gleason score and immediate response to treatment. In these patients, the median serum PSA value was 52.5 $\mu g\ l^{-1}$, and mean Gleason score was 7. Eighty-two per cent of patients had a positive bone scan, and 73% of primary tumours were aneuploid. In addition, the 92 kDa gelatinase activity was expressed in all five patients who did not respond to treatment and died within 6 months from inclusion in the study. Table IV summarises the results. Western blotting, using a non-specific rabbit antiserum prepared against the propeptide of MMP-9, confirmed that 97, 92, 66 and 45 kDa gelatinolytic activities represented different sequences of pro-MMP-9 (Figure 2).

### Discussion

The key step in the natural history of cancer is the malignant transformation of a normal cell. However, the most impor-

**Figure 1** Example of zymograms showing different gelatinolytic bands of activity. Lanes 1 and 2 represent BPH; lanes 3, 4 and 5 represent primary prostatic adenocarcinoma. The 97/92 kDa gelatinase pattern of activity is illustrated.

**Table I** Gelatinolytic matrix metalloproteinase (MMP) degradation bands obtained from tissues of patients with prostatic adenocarcinoma ($n$ = 26) and benign prostatic hyperplasia (BPH, $n$ = 15)

| | *MMP degradation bands ($M_r$, kDa)* | | | | | | |
|---|---|---|---|---|---|---|---|
| | *>100* | *97* | *92* | *72* | *66* | *45* | *29* |
| Carcinoma $n$ = 26 | 14/26 (54%) | 6/26 (23%) | 11/26 (42%) | 20/26 (77%) | 20/26 (77%) | 20/26 (77%) | 5/26 (19%) |
| BPH $n$ = 15 | 3/15 (20%) | 12/15 (80%) | 0/15 (0%) | 4/15 (27%) | 3/15 (20%) | 5/15 (33%) | 0/15 (0%) |
| *Exact P* | *0.05* | *0.0008* | *0.003* | *0.002* | *0.0008* | *0.008* | *0.13* |

*(Critical P value = 0.0042).*

**Table II** Relationship between gelatinolytic matrix metalloproteinase (MMP) degradation bands obtained from tissues of patients with prostatic adenocarcinoma and a positive (CaP BS + ve, $n$ = 14) and negative (CaP BS – ve, $n$ = 12) bone scan

| | *MMP degradation bands ($M_r$, kDa)* | | | | | | |
|---|---|---|---|---|---|---|---|
| | *>100* | *97* | *92* | *72* | *66* | *45* | *29* |
| CaP BS + ve $n$ = 14 | 5/14 (36%) | 4/14 (29%) | 9/14 (64%) | 10/14 (71%) | 11/14 (79%) | 12/14 (86%) | 2/14 (14%) |
| CaP BS – ve $n$ = 12 | 9/12 (75%) | 2/12 (17%) | 2/12 (17%) | 10/12 (83%) | 9/12 (75%) | 8/12 (67%) | 3/12 (25%) |
| *Exact P* | *0.082* | *0.650* | *0.021* | *0.652* | *1.000* | *0.365* | *0.635* |

**Table III** Relationship between gelatinolytic matrix metalloproteinase (MMP) degradation bands and tumour DNA ploidy in 26 patients with prostatic adenocarcinoma

| | *MMP degradation bands* ($M_r$, *kDa*) | | | | | | |
|---|---|---|---|---|---|---|---|
| | *>100* | *97* | *92* | *72* | *66* | *45* | *29* |
| Aneuploid ($n = 11$) | 3/11 (27%) | 2/11 (18%) | 8/11 (73%) | 9/11 (82%) | 7/11 (64%) | 9/11 (82%) | 0/11 (0%) |
| Diploid ($n = 15$) | 11/15 (73%) | 4/15 (27%) | 3/15 (20%) | 11/15 (73%) | 13/15 (87%) | 11/15 (73%) | 5/15 (33%) |

Differences are not statistically significant.

**Table IV** Patients with prostate cancer expressing the 92 kDa metalloproteinase

| *Patient no.* | *Bone scan* | *Ploidy* | *Serum PSA* ($\mu g\ l^{-1}$) | *Gleason score* | *Outcome* |
|---|---|---|---|---|---|
| 1 | + ve | A | 10.0 | 9 | NR |
| 2 | + ve | A | 79.0 | 9 | NR |
| 3 | + ve | A | 61.5 | 7 | A & W |
| 4 | + ve | D | 16.4 | 6 | A & W |
| 5 | + ve | A | 120.0 | 8 | NR |
| 6 | + ve | A | 72.0 | 8 | A & W |
| 7 | + ve | A | 39.0 | 6 | A & W |
| 8 | + ve | A | 120.0 | 7 | NR |
| 9 | + ve | A | 22.0 | 9 | NR |
| 10 | − ve | D | 52.5 | 5 | A & W |
| 11 | − ve | D | 42.0 | 6 | A & W |

NR, non-responder; A & W, alive and well; A, aneuploid tumour; D, diploid tumour; + ve, positive; − ve, negative.

**Figure 2** Example of Western blot profile. Lane 1, BPH; lanes 2 and 3, primary prostatic adenocarcinoma.

tant event in the history of any given tumour is the formation of metastases. It is the ability of a malignant cell to invade and metastasise which determines prognosis; if this is unfavourable, early intervention is necessary to effectively attempt a complete cure. In prostate cancer, metastasis is a major cause of morbidity and mortality (Whitmore, 1984), with many tumours being difficult to treat because of their unpredictable behaviour and the inability of current investigative methods to detect cancers with aggressive and metastatic potential.

Invasion and metastasis are complex and multisequential processes, accomplished by selected subpopulations of malignant cells escaping from the primary tumour (Fidler & Hart, 1982; Fidler, 1991). The ability of tumour cells to migrate across the BM and to degrade extracellular matrix (ECM) occurs through the action of a series of degradative enzymes produced by tumour cells (Tissot *et al.*, 1984; Brown *et al.*, 1990), or following stimulation of host stromal cells to release degradative enzymes by the tumour itself (Basset *et al.*, 1990). Data from animal and experimental studies suggest a 'three-step theory' of tumour invasion (Liotta *et al.*, 1977; Liotta, 1986). The first step involves tumour cell attachment to BM components via specific cell-surface receptors, such as laminin. This is followed by proteolytic enzyme expression by tumour cells to degrade ECM components, particularly type IV collagenases belonging to the MMP family, of which MMP-2 and MMP-9 are specific for type IV collagen. The final step is tumour cell locomotion into the region of the matrix modified by proteolysis.

The MMP family appears to assume increasing importance, in terms of its correlation with the metastatic phenotype in experimental and animal models (Murphy *et al.*, 1989; Cottam & Rees, 1993). Several studies have analysed MMP-2 (72 kDa) and MMP-7 expression in tissue extracts from benign and malignant prostatic tissue, in prostate cancer cell lines and in animal experimental models, demonstrating increased activity in neoplastic compared with benign tissue, and a direct correlation with the invasive phenotype (Pajouh *et al.*, 1991; Boag & Young, 1993; Powell *et al.*, 1993; Stearns & Wang, 1993). However, to our knowledge, the current report is the first to describe a correlation between MMP-9 expression and aggressive prostate cancer. The study represents a novel approach in the search for parameters to define the metastatic potential of prostate cancers. Experimental findings provide an insight into the ability of malignant prostatic and BPH tissue to express gelatinolytic activity potentially involved in tissue invasion. This further suggests that there may be important differences in patterns of enzyme expression between benign and malignant prostatic tissue.

From the results presented, it appears that MMP gelatinolytic enzyme activity detected by zymography is increased in malignant compared with benign prostatic tissue, with the exception of a 97 kDa species. In contrast a 92 kDa gelatinase was not expressed in benign tissue, but was detected in 42% of carcinomas. With adjustments made for multiple comparisons, statistical analysis suggests that the highlighted results are real. Western blotting confirmed that both 97 and 92 kDa activity represent the same enzyme, which in its 97 kDa form in BPH may be represented as a 92 kDa species by activation, once malignant transformation occurs, and processes of invasion and metastasis are in motion, thus exhibiting a reduced molecular weight. In support of this theory, patients who exhibited 92 kDa gelatinase activity *in vitro* had particularly unfavourable clinical parameters, including well-established prognostic factors such as high Gleason scores and serum PSA levels and primary tumour DNA ploidy. Sixty-four per cent of patients with positive bone scans expressed the 92 kDa enzyme, while only 17% of bone scan-negative patients were positive. Furthermore, the five patients in the cancer series studied who did not respond to treatment were all included in the group expressing the 92 kDa band. The 97 kDa gelatinolytic activity may be attributable to a complex between pro-MMP-9 and other proteins, while it is difficult, at this stage of the study, to speculate on the nature of the high molecular weight activity (> 100 kDa) detected in both BPH and carcinomas. This may well represent a different gelatinase, a glycosylation variant or an enzyme complex and remains to be identified, possibly by enzyme purification methods. Detection by Western blotting of further processed lower molecular weight pro-MMP-9 sequence fragments in tumour lysates suggests that tumour but not BPH tissue is capable of processing MMP-9 intracellularly prior to secretion, with possible implications in the activity of the enzyme *in vivo*; this remains to be determined. From the present study, in view of the heterogeneity of the tissue samples analysed and the difficulty in separating prostatic cancer cells from the surrounding stroma and fibroblasts, it is impossible to assume that all the enzymes detected were produced solely by tumour cells. Indeed, it has been shown from work on breast cancer and in skin tumours that stromal cells surrounding tumour foci can be the main source of proteolytic enzyme synthesis (Basset *et al.*, 1990; Karelina *et al.*, 1993). This may well be the case in the work presented herein, and can only be determined by further studies using *in situ* hybridisation and immunohistochemistry. Furthermore, the gelatinase activity was detected *in vitro*, and may not necessarily represent *in vivo* behaviour, which could be affected in particular by specific protease inhibitors, including TIMP-1 and TIMP-2. This may constitute important further work to validate the present findings in the *in vivo* situation.

The 92 kDa pro-MMP-9 is an immunologically distinct glycosylated enzyme, mainly expressed by monocytes, macrophages and polymorphonuclear leucocytes in the presence of inflammation, and by tumour cells (Yamagata *et al.*, 1988, 1989; Murphy *et al.*, 1989). It is possible that the increased 92 kDa expression found in malignant prostatic samples investigated in this study is merely the reflection of increased inflammation in tumour tissue compared with BPH. However, histopathological examination of representative tissue samples subjected to zymography did not consistently reveal the presence of prominent inflammatory infiltrates in all the specimens analysed. The 92 kDa enzyme is also known to be frequently expressed by malignant tumours (Pyke *et al.*, 1992; 1992; Sato *et al.*, 1992), and can be induced by *ras* oncogene transfection (Ballin *et al.*, 1988). It has been correlated with increasing metastatic potential and invasive behaviour in a number of malignant cell types (Yamagata *et al.*, 1988; Sato & Seiki, 1993), and with the metastatic phenotype in transformed rat embryo cells (Bernhard *et al.*, 1990). Data from these studies largely support the present findings, suggesting a possible correlation between 92 kDa MMP expression and aggressive prostate cancers.

Results obtained from this study warrant further investigation into the potential use of differential MMP expression as new prognostic markers in prostate cancer, and as possible predictor of clinical response to treatment. Further work extending the present investigation to cellular and molecular levels will further our understanding of the ubiquitous biology of prostatic adenocarcinoma. Such studies may, in future, identify a group of prostate cancer patients whose tumours carry definite metastatic potential. This would enable aggressive treatment to be directed at those most at risk of developing progressive disease, in an attempt to decrease the morbidity and mortality associated with this common malignancy.

This study was supported by a grant from the Trustees for the Former United Sheffield Hospitals, and by the Yorkshire Cancer Research Campaign.

### References

BALLIN, M., GOMEZ, D.E., SINHA, C.C. & THORGEIRSSON, U.P. (1988). Ras oncogene mediated induction of a 92 kDa metalloproteinase; strong correlation with the malignant phenotype. *Biochem. Biophys. Res. Commun.*, **154**, 832–838.

BASSET, P., BELLOCQ, J.P., WOLF, C., STOLL, I., HUTIN, P., LIMACHER, J.M., PODHAJCER, O.L., CHENARD, M.P., RIO, M.C. & CHAMBON, P. (1990). A novel metalloproteinase gene specifically expressed in stromal cells of breast carcinomas. *Nature*, **348**, 699–704.

BERNHARD, E.J., MUSCHEL, R.J. & HUGHES, E.N. (1990). $M_r$ 92,000 gelatinase release correlates with the metastatic phenotype in transformed rat embryo cells. *Cancer Res.*, **50**, 3872–3877.

BOAG, A.H. & YOUNG, I.D. (1993). Immunohistochemical analysis of type IV collagenase expression in prostatic hyperplasia and adenocarcinoma. *Mod. Pathol.*, **6**, 65–68.

BROWN, P.D., LEVY, A.T., MARGULIES, M.K. & LIOTTA, L.A. (1990). Independent expression and cellular processing of $M_r$ 72,000 type IV collagenase and interstitial collagenase in tumorigenic cell lines. *Cancer Res.*, **50**, 6184–6191.

COTTAM, D.W. & REES, R.C. (1993). Regulation of matrix metalloproteinases: their role in tumor invasion and metastasis (review). *Int. J. Oncol.*, **2**, 861–872.

DEVERE WHITE, R.W. & DEITCH, A.D. (1989). Flow cytometry in prostatic cancer. In *The Prostate*, Fitzpatrick, J.M. & Krane, R.J. (eds) pp. 307–314. Churchill Livingstone: Edinburgh.

FIDLER, I.J. (1991). Cancer metastasis. *Br. Med. Bull.*, **47**, 157–177.

FIDLER, I.J. & HART, I.R. (1982). Biological diversity in metastatic neoplasms: origins and implications. *Science*, **217**, 998–1003.

GEORGE, N.J.R. (1988). Natural history of localized prostatic cancer managed by conservative therapy alone. *Lancet*, **i**, 494–497.

GITTES, R.F. (1991). Carcinoma of the prostate. *N. Engl. J. Med.*, **4**, 236–245.

GLEASON, D.F. (1966). Classification of prostatic carcinoma. *Cancer Chemother. Rep.*, **50**, 125–128.

GLEASON, D.F. & MELLINGER, G.T. (1974). Prediction of prognosis for prostatic adenocarcinoma by combined histological grading and clinical staging. *J. Urol.*, **111**, 58–64.

HEUSSEN, C. & DOWDLE, E.B. (1980). Electrophoretic analysis of plasminogen activators in polyacrylamide gels containing sodium dodecyl sulphate and copolymerized substrates. *Anal. Biochem.*, **102**, 196–202.

HIRST, Jr, A.E. & BERGMAN, R.T. (1954). Carcinoma of the prostate in men 80 or more years old. *Cancer*, **7**, 136–141.

JOHANSSON, J.E., ADAMI, H.O., ANDERSSON, S.O., BERGSTROM, R., KRUSEMO, U.B. & KRAAZ, W. (1989). Natural history of localized prostatic cancer: a population based study in 223 untreated patients. *Lancet*, **i**, 799–803.

JOHANSSON, J.E., ADAMI, H.O., ANDERSSON, S.O., BERSTRÖM, R., HOLMBERG, L. & KRUSEMO, U.B. (1992). High 10-year survival rate in patients with early, untreated prostate cancer. *JAMA*, **267**, 2191–2196.

KARELINA, T.V., HRUZA, G.J., GOLDBERG, G.I. & EISEN, A.Z. (1993). Localization of 92-kDa Type IV collagenase in human skin tumors: comparison with normal human fetal and adult skin. *J. Invest. Dermatol.*, **100**, 159–165.

LAEMMLI, U.K. (1970). Cleavage of structural proteins during the assembly of the head of bacteriophage T4. *Nature*, **227**, 680–685.

LIOTTA, L.A. (1986). Tumour invasion and metastases. Role of the extracellular matrix: Rhoads Memorial Award Lecture. *Cancer Res.*, **46**, 1–7.

LIOTTA, L.A. & STETLER-STEVENSON, W.G. (1991). Tumour invasion and metastasis, and imbalance of positive and negative regulation. *Cancer Res.*, **51**, 5064s–5059s.

LIOTTA, L.A., KLEINERMAN, J., CATANZARA, P. & RYNBRANDT, D. (1977). Degradation of basement membrane by murine tumor cells. *J. Natl Cancer Inst.*, **58**, 1427–1439.

LIOTTA, L.A., STEEG, P.S. & STETLER-STEVENSON, W.G. (1991). Cancer metastasis and angiogenesis: an imbalance of positive and negative regulation. *Cell*, **64**, 327–336.

MATRISIAN, L.M. (1990). Metalloproteinases and their inhibitors in matrix remodelling. *Trends Genet.*, **6**, 121–125.

MURPHY, G., REYNOLDS, J.J. & HEMBRY, R.M. (1989). Metalloproteinases and cancer invasion and metastasis. *Int. J. Cancer*, **44**, 757–760.

MURPHY, G.J., MURPHY, G. & REYNOLDS, J.J. (1991). The origin of matrix metalloproteinases and their familial relationship. *FEBS Lett.*, **289**, 4-7.

OFFICE OF POPULATION CENSUSES AND SURVEYS (1985). *Cancer Statistics, England and Wales*. Series MBI, No. 18. HMSO: London.

PAJOUH, M.S., NAGLE, R.B., BREATNACH, R., FINCH, J.S., BRAWER, M.K. & BOWDEN, G.T. (1991). Expression of metalloproteinase genes in human prostate cancer. *J. Cancer Res. Clin. Oncol.*, **117**, 144-150.

POWELL, W.C., KNOX, J.D., NAVRE, M., GROGAN, T.M., KITTELSON, J., NAGLE, R.B. & BOWDEN, G.T. (1993). Expression of the Metalloproteinase Matrilysin in DU-145 cells increases their invasive potential in severe combined immunodeficient mice. *Cancer Res.*, **53**, 417-422.

PYKE, C., RALFKIGER, E., HUHTALA, P., HURSKAINEN, T., DANØ, K. & TRYGGVASON, K. (1992). Localization of messenger RNA for $M_r$ 72,000 and 92,000 type IV collagenases in human skin cancers by *in situ* hybridization. *Cancer Res.*, **52**, 1336-1341.

SATO, H. & SEIKI, M. (1993). Regulatory mechanism of 92 kDa type IV collagenase gene expression which is associated with invasiveness of tumor cells. *Oncogene*, **8**, 395-405.

SATO, H., KIDA, Y., MAI, M., ENDO, Y., SASAKI, T., TANAKA, J. & SEIKI, M. (1992). Expression of genes encoding type IV collagen degrading metalloproteinases and tissue inhibitors of metalloproteinases in various tumour cells. *Oncogene*, **7**, 77-85.

SCHWEDER, T. & SPJOTVOLL, E. (1982). Plots of p values to evaluate many tests simultaneously. *Biometrika*, **69**, 493-502.

STEARNS, M.E. & WANG, M. (1993). Type IV collagenase (Mr 72,000) expression in human prostate: benign and malignant tissue. *Cancer Res.*, **53**, 878-883.

STETLER-STEVENSON, W.G., KRUTZSCH, H.C., WACHER, M.P., MARGULIES, I.M. & LIOTTA, L.A. (1989). The activation of human type IV collagenase proenzyme. Sequence identification of the major conversion product following organomercurial activation. *J. Biol. Chem.*, **264**, 1353-1356.

TAVARES, A.S., COSTA, J. & COSTA MAIA, J. (1973). Correlation between ploidy and prognosis in prostatic carcinoma. *J. Urol.*, **109**, 676-679.

TISSOT, J.D., HAVERT, J. & BACHMANN, F. (1984). Characterization of plasminogen activators from normal human breast and colon and from breast and colon carcinoma. *Int. J. Cancer*, **34**, 295-302.

TOWBIN, H., STAEHELIN, T. & GORDON, J. (1979). Electrophoretic transfer of proteins from polyacrylamide gels to nitrocellulose sheets: procedure and some applications. *Proc. Natl Acad. Sci. USA*, **76**, 4350-4354.

WANG, C.-S. & SMITH, R.L. (1975). Lowry determination of protein in the presence of Triton X-100. *Anal. Biochem.*, **63**, 414-417.

WHITMORE, Jr, W.F. (1984). Natural history and staging of prostate cancer. *Urol. Clin. N. Am.*, **11**, 205-220.

WILSON, M.J., NORRIS, H., KAPOOR, D., WOODSON, M., LIMAS, C. & SINHA, A.A. (1993). Gelatinolytic and caseinolytic proteinase activities in human prostatic secretions. *J. Urol.*, **149**, 653-658.

WOESSNER, Jr, F.J. (1991). Matrix metalloproteinases and their inhibitors in connective tissue remodelling. *FASEB J.*, **5**, 2145-2154.

YAMAGATA, S., ITO, Y., TANAKA, R. & SCHIMIZU, S. (1988). Gelatinases of metastatic cell line of murine colonic carcinoma as detected by substrate-gel electrophoresis. *Biochem. Biophys. Res. Commun.*, **151**, 158-162.

YAMAGATA, S., TANAKA, R., ITO, Y., SCHIMIZU, S. (1989). Gelatinases of murine metastatic tumor cells. *Biochem. Biophys. Res. Commun.*, **158**, 228-234.


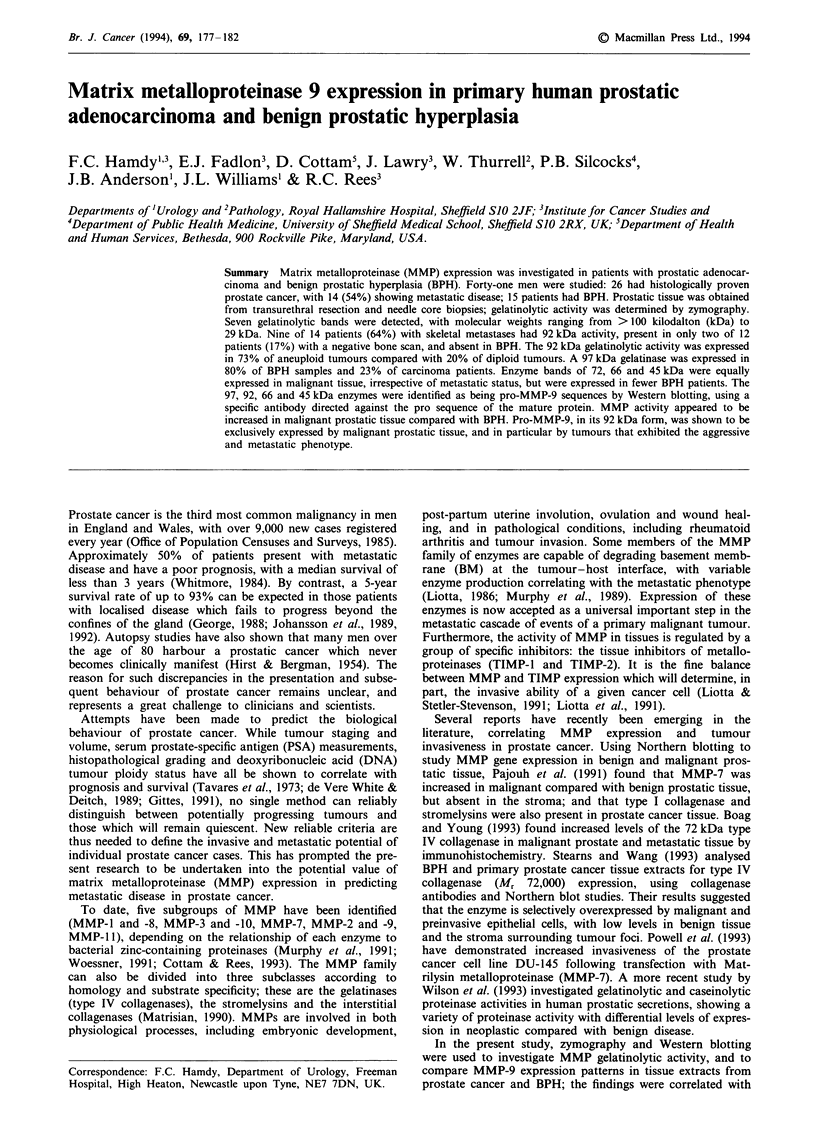

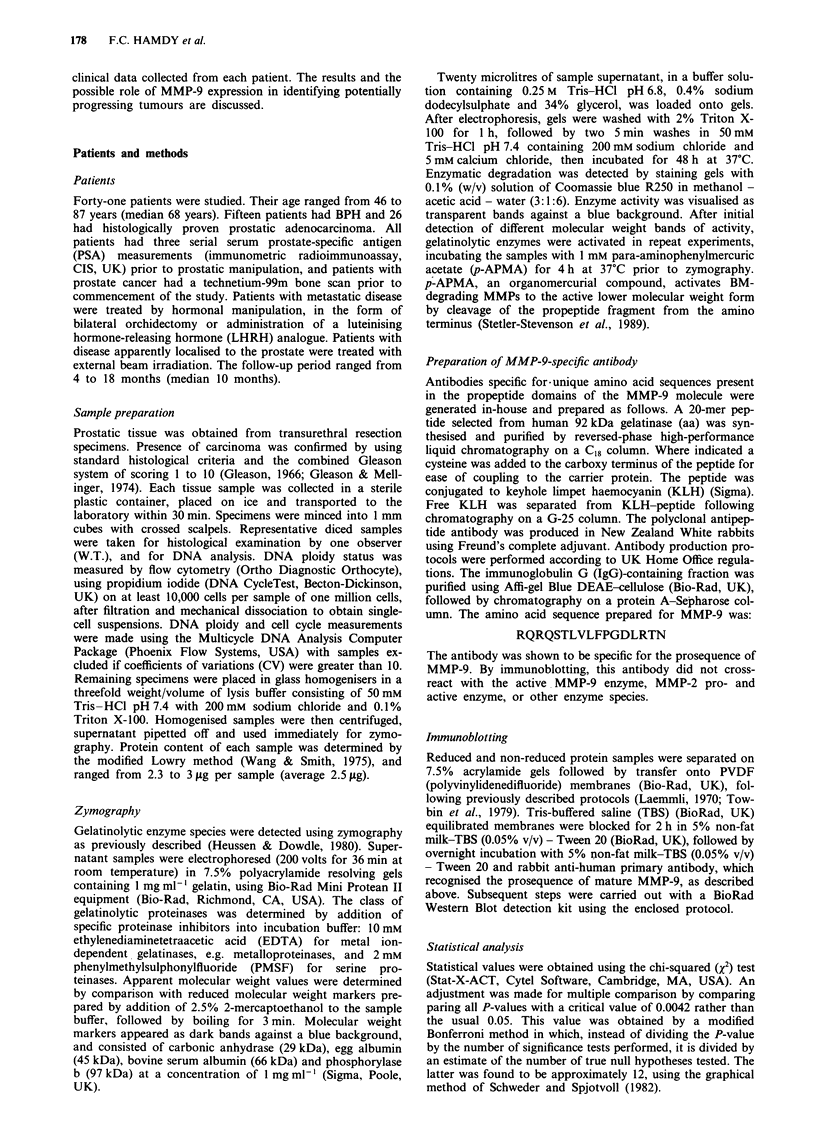

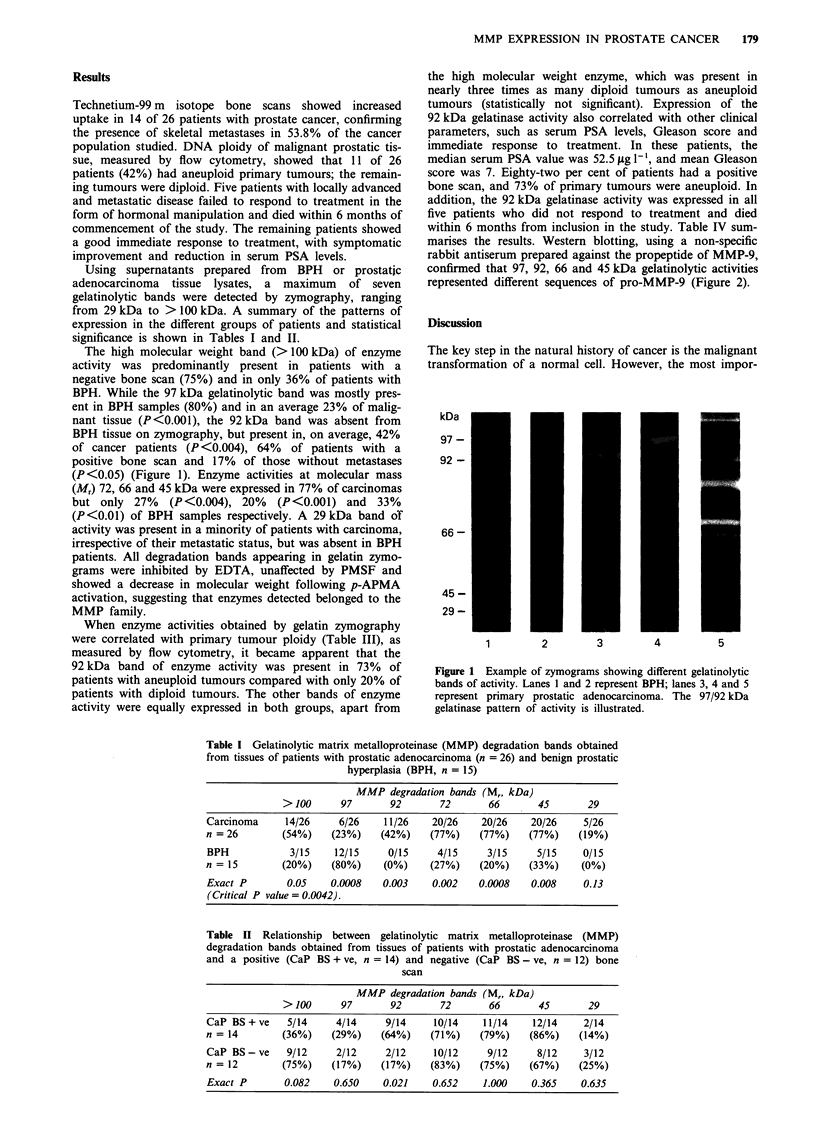

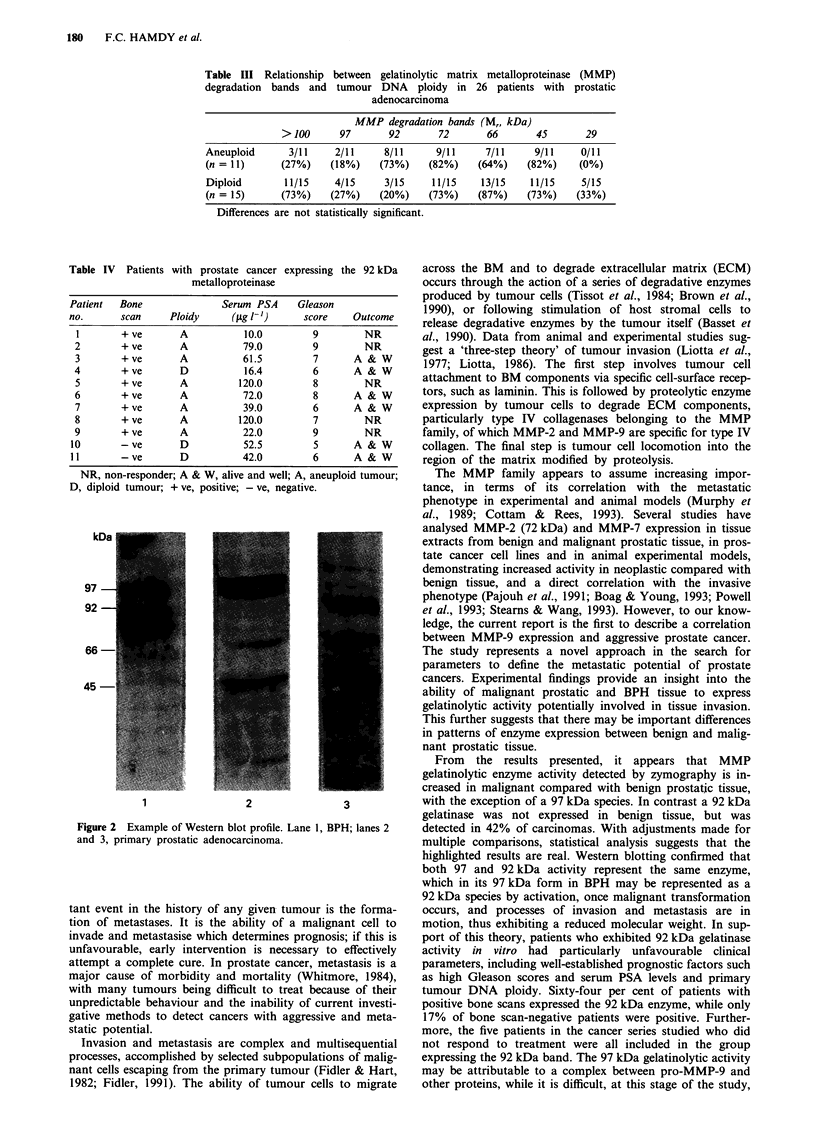

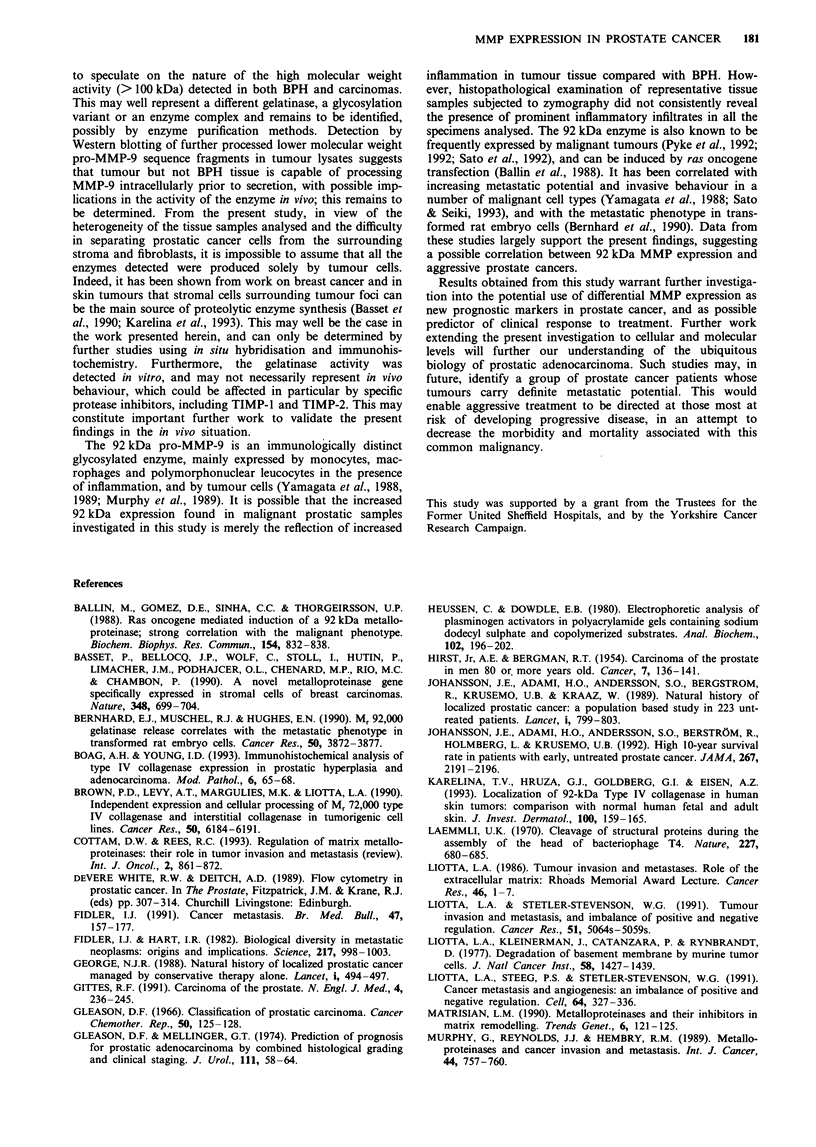

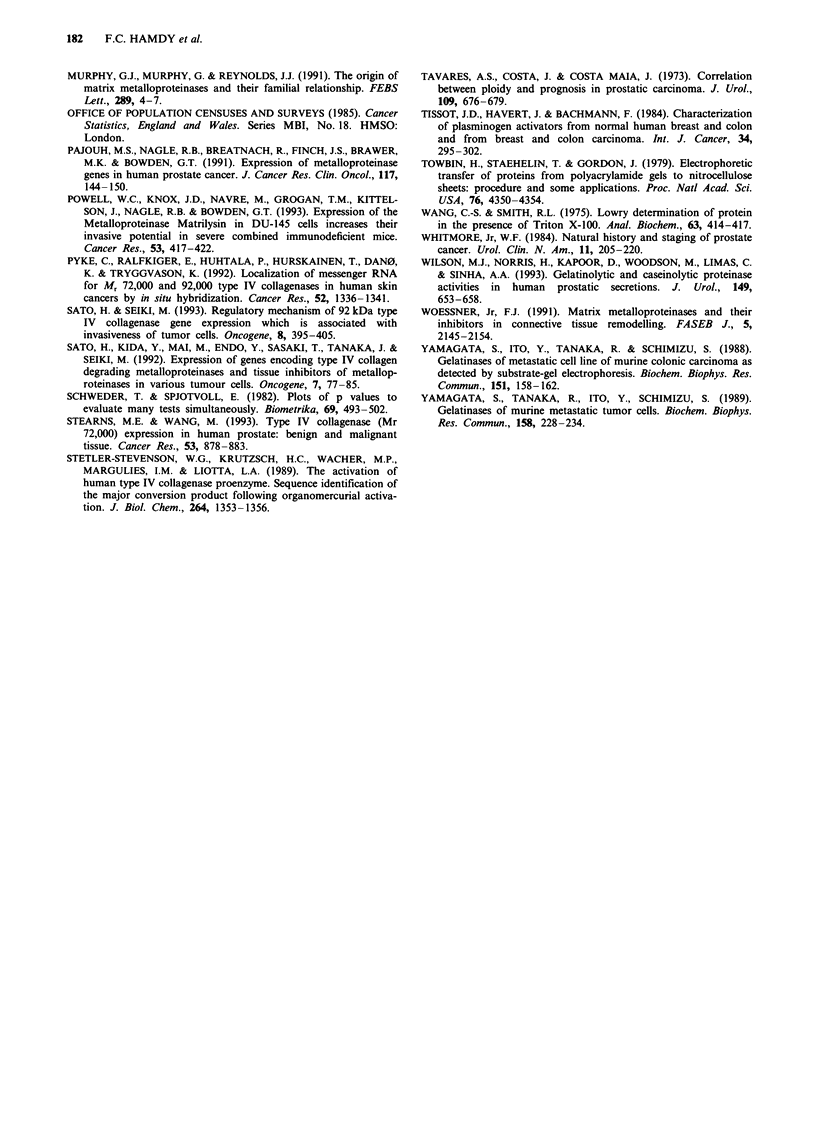


## References

[OCR_00610] Ballin M., Gomez D. E., Sinha C. C., Thorgeirsson U. P. (1988). Ras oncogene mediated induction of a 92 kDa metalloproteinase; strong correlation with the malignant phenotype.. Biochem Biophys Res Commun.

[OCR_00616] Basset P., Bellocq J. P., Wolf C., Stoll I., Hutin P., Limacher J. M., Podhajcer O. L., Chenard M. P., Rio M. C., Chambon P. (1990). A novel metalloproteinase gene specifically expressed in stromal cells of breast carcinomas.. Nature.

[OCR_00623] Bernhard E. J., Muschel R. J., Hughes E. N. (1990). Mr 92,000 gelatinase release correlates with the metastatic phenotype in transformed rat embryo cells.. Cancer Res.

[OCR_00628] Boag A. H., Young I. D. (1993). Immunohistochemical analysis of type IV collagenase expression in prostatic hyperplasia and adenocarcinoma.. Mod Pathol.

[OCR_00633] Brown P. D., Levy A. T., Margulies I. M., Liotta L. A., Stetler-Stevenson W. G. (1990). Independent expression and cellular processing of Mr 72,000 type IV collagenase and interstitial collagenase in human tumorigenic cell lines.. Cancer Res.

[OCR_00649] Fidler I. J. (1991). Cancer metastasis.. Br Med Bull.

[OCR_00653] Fidler I. J., Hart I. R. (1982). Biological diversity in metastatic neoplasms: origins and implications.. Science.

[OCR_00657] George N. J. (1988). Natural history of localised prostatic cancer managed by conservative therapy alone.. Lancet.

[OCR_00661] Gittes R. F. (1991). Carcinoma of the prostate.. N Engl J Med.

[OCR_00665] Gleason D. F. (1966). Classification of prostatic carcinomas.. Cancer Chemother Rep.

[OCR_00669] Gleason D. F., Mellinger G. T. (1974). Prediction of prognosis for prostatic adenocarcinoma by combined histological grading and clinical staging.. J Urol.

[OCR_00680] HIRST A. E., BERGMAN R. T. (1954). Carcinoma of the prostate in men 80 or more years old.. Cancer.

[OCR_00674] Heussen C., Dowdle E. B. (1980). Electrophoretic analysis of plasminogen activators in polyacrylamide gels containing sodium dodecyl sulfate and copolymerized substrates.. Anal Biochem.

[OCR_00690] Johansson J. E., Adami H. O., Andersson S. O., Bergström R., Holmberg L., Krusemo U. B. (1992). High 10-year survival rate in patients with early, untreated prostatic cancer.. JAMA.

[OCR_00684] Johansson J. E., Adami H. O., Andersson S. O., Bergström R., Krusemo U. B., Kraaz W. (1989). Natural history of localised prostatic cancer. A population-based study in 223 untreated patients.. Lancet.

[OCR_00696] Karelina T. V., Hruza G. J., Goldberg G. I., Eisen A. Z. (1993). Localization of 92-kDa type IV collagenase in human skin tumors: comparison with normal human fetal and adult skin.. J Invest Dermatol.

[OCR_00702] Laemmli U. K. (1970). Cleavage of structural proteins during the assembly of the head of bacteriophage T4.. Nature.

[OCR_00717] Liotta L. A., Kleinerman J., Catanzaro P., Rynbrandt D. (1977). Degradation of basement membrane by murine tumor cells.. J Natl Cancer Inst.

[OCR_00722] Liotta L. A., Steeg P. S., Stetler-Stevenson W. G. (1991). Cancer metastasis and angiogenesis: an imbalance of positive and negative regulation.. Cell.

[OCR_00712] Liotta L. A., Stetler-Stevenson W. G. (1991). Tumor invasion and metastasis: an imbalance of positive and negative regulation.. Cancer Res.

[OCR_00707] Liotta L. A. (1986). Tumor invasion and metastases--role of the extracellular matrix: Rhoads Memorial Award lecture.. Cancer Res.

[OCR_00727] Matrisian L. M. (1990). Metalloproteinases and their inhibitors in matrix remodeling.. Trends Genet.

[OCR_00738] Murphy G. J., Murphy G., Reynolds J. J. (1991). The origin of matrix metalloproteinases and their familial relationships.. FEBS Lett.

[OCR_00731] Murphy G., Reynolds J. J., Hembry R. M. (1989). Metalloproteinases and cancer invasion and metastasis.. Int J Cancer.

[OCR_00748] Pajouh M. S., Nagle R. B., Breathnach R., Finch J. S., Brawer M. K., Bowden G. T. (1991). Expression of metalloproteinase genes in human prostate cancer.. J Cancer Res Clin Oncol.

[OCR_00756] Powell W. C., Knox J. D., Navre M., Grogan T. M., Kittelson J., Nagle R. B., Bowden G. T. (1993). Expression of the metalloproteinase matrilysin in DU-145 cells increases their invasive potential in severe combined immunodeficient mice.. Cancer Res.

[OCR_00761] Pyke C., Ralfkiaer E., Huhtala P., Hurskainen T., Danø K., Tryggvason K. (1992). Localization of messenger RNA for Mr 72,000 and 92,000 type IV collagenases in human skin cancers by in situ hybridization.. Cancer Res.

[OCR_00772] Sato H., Kida Y., Mai M., Endo Y., Sasaki T., Tanaka J., Seiki M. (1992). Expression of genes encoding type IV collagen-degrading metalloproteinases and tissue inhibitors of metalloproteinases in various human tumor cells.. Oncogene.

[OCR_00767] Sato H., Seiki M. (1993). Regulatory mechanism of 92 kDa type IV collagenase gene expression which is associated with invasiveness of tumor cells.. Oncogene.

[OCR_00782] Stearns M. E., Wang M. (1993). Type IV collagenase (M(r) 72,000) expression in human prostate: benign and malignant tissue.. Cancer Res.

[OCR_00787] Stetler-Stevenson W. G., Krutzsch H. C., Wacher M. P., Margulies I. M., Liotta L. A. (1989). The activation of human type IV collagenase proenzyme. Sequence identification of the major conversion product following organomercurial activation.. J Biol Chem.

[OCR_00794] Tavares A. S., Costa J., Maia J. C. (1973). Correlation between ploidy and prognosis in prostatic carcinoma.. J Urol.

[OCR_00799] Tissot J. D., Hauert J., Bachmann F. (1984). Characterization of plasminogen activators from normal human breast and colon and from breast and colon carcinomas.. Int J Cancer.

[OCR_00805] Towbin H., Staehelin T., Gordon J. (1979). Electrophoretic transfer of proteins from polyacrylamide gels to nitrocellulose sheets: procedure and some applications.. Proc Natl Acad Sci U S A.

[OCR_00811] Wang C., Smith R. L. (1975). Lowry determination of protein in the presence of Triton X-100.. Anal Biochem.

[OCR_00814] Whitmore W. F. (1984). Natural history and staging of prostate cancer.. Urol Clin North Am.

[OCR_00818] Wilson M. J., Norris H., Kapoor D., Woodson M., Limas C., Sinha A. A. (1993). Gelatinolytic and caseinolytic proteinase activities in human prostatic secretions.. J Urol.

[OCR_00824] Woessner J. F. (1991). Matrix metalloproteinases and their inhibitors in connective tissue remodeling.. FASEB J.

[OCR_00829] Yamagata S., Ito Y., Tanaka R., Shimizu S. (1988). Gelatinases of metastatic cell lines of murine colonic carcinoma as detected by substrate-gel electrophoresis.. Biochem Biophys Res Commun.

[OCR_00835] Yamagata S., Tanaka R., Ito Y., Shimizu S. (1989). Gelatinases of murine metastatic tumor cells.. Biochem Biophys Res Commun.

